# Attention impairments and ADHD symptoms in adult narcoleptic patients with and without hypocretin deficiency

**DOI:** 10.1371/journal.pone.0182085

**Published:** 2017-08-01

**Authors:** Marco Filardi, Fabio Pizza, Lorenzo Tonetti, Elena Antelmi, Vincenzo Natale, Giuseppe Plazzi

**Affiliations:** 1 DIBINEM – Department of Biomedical and Neuromotor Sciences, University of Bologna, Bologna, Italy; 2 IRCCS – Istituto delle Scienze Neurologiche, AUSL di Bologna, Bologna, Italy; 3 Department of Psychology, University of Bologna, Bologna, Italy; Charité - Universitätsmedizin Berlin, GERMANY

## Abstract

**Background:**

Attentional complaints are common in narcolepsy patients and can overlap with daytime sleepiness features. Few studies attempted to characterize attentional domains in narcolepsy leading to controversial results. We aimed to assess the impact of hypocretin deficiency on attentional functioning by comparing performances on the attention network test (ANT) of narcoleptic patients with hypocretin deficiency (narcolepsy type 1—NT1) versus patients without hypocretin deficiency (narcolepsy type 2—NT2) and healthy controls. We also addressed frequency and severity of psychopathological symptoms and their influence on performances on ANT.

**Methods:**

Twenty-one NT1 patients, fifteen NT2 patients and twenty-two healthy controls underwent the ANT, which allows assessing three separate attentional processes (alerting, orienting and executive control), and a psychometric assessment including questionnaires on attention-deficit hyperactivity disorder (ADHD), obsessive-compulsive disorder, anxiety and depression symptoms.

**Results:**

NT1 and NT2 patients presented with slower reaction times compared to controls. NT1 patients exhibited an impairment of alerting network relative to NT2 and healthy controls, while orienting and executive control networks efficiency were comparable between groups. NT1 and NT2 displayed higher severity of ADHD inattentive domain than controls, NT1 patients also displayed higher severity of ADHD hyperactive domain and depressive symptoms. In NT1, ADHD and depressive symptoms were positively correlated.

**Conclusions:**

Despite a shared slowing of reaction times in both NT1 and NT2, a selective impairment of alerting network was present only in hypocretin deficient patients. Clinicians should carefully consider attentional deficits and psychopathological symptoms, including ADHD symptoms, in the clinical assessment and management of patients with narcolepsy.

## Introduction

Narcolepsy type 1 (NT1) and narcolepsy type 2 (NT2) are two central disorders of hypersomnolence characterized by chronic hypersomnolence with sleep onset typically showing a rapid transitions into rapid eye movement (REM) sleep, along with additional untimely manifestations of dissociated REM sleep (hypnagogic/hypnopompic hallucinations, sleep paralysis) [1]. NT1, moreover, is characterized by cataplexy (sudden loss of muscle tone during wakefulness, triggered by emotions), and has a firmly established pathophysiology linked to the loss of hypothalamic hypocretin-producing neurons [1,2]. This deficit was shown in post-mortem studies and is mirrored by reduced or absent hypocretin-1 (Hcrt-1) levels in the cerebrospinal fluid (CFS), making NT1 a hypocretin deficiency syndrome [3,4]. Conversely, in NT2 the hypocretinergic system is intact or only partially compromised and cataplexy is absent [1,4].

The hypocretinergic system modulates a wide variety of behavioral and physiological processes, beyond sleep regulation [5]: these findings led some researchers to question whether an underlying cognitive impairment might accompany the classical symptoms of NT1. Concentration and memory problems are indeed commonly reported by NT1 patients [6], however the characterization of these deficits in experimental settings has been elusive [7].

Objective evaluations of memory performance with standardized neuropsychological batteries showed unimpaired short-term memory and only mild deficit in long-term memory [8,9], while other studies have pointed to an impairment of sleep-dependent memory consolidation processes in narcolepsy [10,11]. By contrast, several studies highlighted deficits in the attentional domains [7].

NT1 patients consistently exhibited deficits of sustained attention, especially while performing long and monotonous tasks [12,13], and markedly slower and more variable reaction times (RTs) with preserved accuracy in shorter tasks [9,14,15]. The presence of deficit in specific attentional subcomponents is more controversial and possibly related to the neuropsychological tasks adopted.

A way to overcome this issue is to select cognitive tasks based on neurocognitive models of attentional functioning. According to an influential model the source of attention involves a specific set of anatomical areas organized into three networks: the alerting, orienting, and executive control networks [16]. Each network displays distinct, although partially overlapping, neuroanatomical structures and is modulated by a dominant neurotransmitter [17,18]. The attention network test (ANT) assesses the functioning of these networks with a single, short, task [19].

Parallel to cognitive difficulties, narcolepsy patients also display high rates of psychiatric comorbidity, most notably anxiety and depression disorders [20,21]. Recent evidence also highlighted an association between childhood attention-deficit/hyperactivity disorder (ADHD) and narcolepsy: in a cross-sectional study 35.3% of NT2 and 19.7% of NT1 children showed clinically significant ADHD symptoms compared to 4.8% of controls [22]. Similar results emerged in a retrospective study on adult narcoleptics in which 37% of patients reported symptoms suggestive of childhood ADHD [23]. Surprisingly, data on the persistence of ADHD symptoms in narcoleptic adults are still lacking. Our study aims were therefore the following: (1) to investigate whether changes in attention networks functioning could be observed in NT1 compared to NT2 and healthy controls; (2) to assess frequency and severity of ADHD, obsessive-compulsive disorder (OCD), anxiety and depression symptoms in narcoleptic patients versus healthy controls; and (3) to correlate attentional performances with disease severity and psychological traits.

## Material and methods

### 2.1 Participants

Participants were consecutive adult patients evaluated for complaints of chronic hypersomnolence at the outpatient clinic for Narcolepsy of the Department of Biomedical and Neuromotor Sciences, University of Bologna, and who received a final diagnosis of narcolepsy (either NT1 or NT2) according to current International Classification of Sleep Disorders 3^rd^ edition (ICSD-3) criteria [1].

Patients underwent our standardized diagnostic protocol encompassing clinical evaluation with assessment of subjective sleepiness by means of Epworth Sleepiness Scale (ESS) [24], cerebral magnetic resonance imaging, hospitalization with 48-hr continuous polysomnographic recording, Multiple Sleep Latency Test (MSLT), cataplexy video-documentation, human leukocyte antigen (HLA) typing and lumbar puncture for CSF Hcrt-1 assay [25]. The final study sample included 36 patients, 21 NT1 (9 males, age 36.19 ± 11.94 y) and 15 NT2 (6 males, age 35.53 ± 12.95 y). All patients were drug-naïve on hospitalization, none had a previously diagnosed sleep or psychiatric disorder, and had a long-lasting overlooked disease history (namely, 15 ± 9 years for NT1 patients and 16 ± 14 years for NT2 patients) [26]. NT1 patients presented with severe daytime sleepiness (mean ESS = 15.90 ± 5.31) and documented cataplexy (*n* = 21/21). NT2 patients presented with severe daytime sleepiness (mean ESS = 15.53 ± 5.45), absence of cataplexy on clinical interview and in-laboratory assessment, and mean sleep latency < 8 min with at least two SOREMPs on MSLT or nocturnal polysomnography, the latter excluding other concomitant sleep disorders [1].

Twenty-two age- and sex-matched healthy controls (11 males, age 34.95 ± 11.52 y) were recruited from the local community. Inclusion criteria were: (a) ability to understand and provide informed consent; (b) no history of neurological or psychiatric disorder; and (c) no current intake of medications known to alter sleep or cognition.

The study was approved by the Comitato Etico Interaziendale Bologna-Imola and all participants provided written informed consent.

### 2.2 Attention network test

The original version of the ANT was used in this study, for a detailed description of the task we refer the reader to the paper by Fan and co-authors [19].

We considered the use of the ANT to characterize attentional functioning in narcolepsy patients for several reasons. First, it is a brief but very informative task as it provides information on three different types of attentional processes, thus being a suitable tool for studying patients suffering from a remarkable time-on-task effect [7,27]. Second, it is a complex task that involves attention control and executive functions, cognitive domains that are required to properly perform daily life activities. Third, it was developed according to a widely accepted neurocognitive model of attention functioning and the neural correlates of each networks have been reliably identified by means of neuroimaging and neurochemical techniques [17,18].

Fourth, the cognitive tasks most commonly used to investigate attentional functioning in central disorders of hypersomnolence patients (i.e. sustained attention to response task; continuous performance task; psychomotor vigilance task; test of variables of attention) focus on vigilance/sustained attention: participants have to wait, over a long time period, the appearance of an infrequent target. The main task outcome is not RTs but error score, namely omission errors (target appears and subject does not respond) and commission errors (participant responds but the target did not appear); accordingly, these tasks can highlight a general attention deficit but do not allow to investigating the features of this deficit, while the ANT can overcome this limitation.

NT1 and NT2 patients were evaluated during diagnostic hospitalization over two days. On the first day participants were instructed on the study protocol, provided informed consent, and practiced with the task. On the second day, the test was administered at fixed hour (17:30) based on previous evidence, in healthy subjects, of a circadian modulation of attention networks efficiency [28].

Healthy controls underwent the same experimental protocol and were evaluated at the Laboratory of Applied Chronopsychology, Department of Psychology, University of Bologna.

Night-sleep, daytime-naps duration and quality were monitored in both patients (by means of nighttime PSG in the night prior the testing session) and healthy controls (by means of actigraphic monitoring), prior to the administration of the ANT. Participants were invited to take a brief (maximum 30 min) nap before performing the ANT, if they felt sleepy.

### 2.3 Self-report measures

Upon completing the ANT participants filled in questionnaires assessing symptoms of ADHD, OCD, depression and anxiety. The Adult ADHD Self-Report Scale Symptom Checklist (ASRS-v1.1) is a questionnaire that assesses the severity of ADHD symptoms in adulthood [29]. The ASRS provides two subscores: the first (ASRS inattentive score, ASRS_In_) assesses inattentive symptoms (i.e. difficulties to pay attention, excessive distractibility, difficulties organizing tasks), and the second (ASRS hyperactive score, ASRS_Hy_) assesses hyperactive/impulsive symptoms (i.e. fidget or restless behavior, excessive activity, difficulty in remaining seated). Another score computed on six items (ASRS Screener, ASRS_Scr_) provides clinical cut-off values [30].

The Obsessive Compulsive Inventory-Revised (OCIr) is an 18-item questionnaire assessing the degree of distress associated with obsessions and compulsions. Participants were asked to express, on a 5-point Likert scale (ranging from 0 = not at all to 4 = extremely), the distress associated with the proposed statements [31].

The Beck Depression Inventory (BDI) and the State-Trait Anxiety Inventory (STAI) were used to assess depression and anxiety [32,33] respectively; circadian typology was evaluated with the reduced version of morningness/eveningness questionnaire (rMEQ) [34].

### 2.4 Statistical analyses

Data were explored with descriptive statistics (mean ± SD). Group differences in demographic, clinical data, and questionnaire scores were analyzed with chi-square test, Mann-Whitney U test, and one-way analysis of variance (ANOVA).

For ANT measures, we computed mean overall RTs, accuracy rate, and efficiency of attention networks. Trials in which participants made errors and trials with RTs ± 3 SD from the mean were excluded from analysis [19]. To avoid biases related to slower RTs in patients, we computed a proportional transformation on network score to examine effects independently of global slowing; proportional scores (Pro) were calculated by dividing networks' score for a measure of the information processing speed (RT_All_ = mean RTs for all 12 warning Cue X flanker conditions) [35].

Thereafter, we performed an ANOVA considering the following variables: overall RTs, accuracy rate, alerting, orienting and executive network absolute and proportional scores. Bonferroni post-hoc were used to determine the nature of the between-group differences, partial eta-squared (*η*^2^_*p*_) was computed as a measure of effect size, with values of .14, .06 and .01 indicating respectively a large, medium or small effect [36]. Furthermore, for each attention network we performed a mixed ANOVA with group as between-subject factor (3 levels) and the type of cue or flanker that defined the specific network as within-subject factor (2 levels, i.e. no-cue and double-cue for alerting, central-cue and spatial-cue for orienting, incongruent and congruent flanker for executive).

Finally, the relationship between clinical, self-reported measures and attention networks scores was explored, separately for each group, with Pearson correlation coefficient analysis. Statistical analyses were conducted using SPSS 19.0 (SPSS, Inc. Chicago, Ill). Results with p values <0.05 were considered statistically significant.

## Results

### 3.1 Participant characteristics and self-reported measures

Demographic, clinical, neurophysiological characteristics and questionnaires' scores are reported in Table 1. Chi-square and one-way ANOVA showed no group differences in either sex or age, as well as in chronotype distribution. As expected, NT1 and NT2 displayed a higher level of subjective sleepiness than controls, and NT1 also displayed higher BMI than NT2 and controls.

**Table 1:** Demographic, clinical, neurophysiological characteristics and questionnaire score of patients with narcolepsy type 1 (NT1), narcolepsy type 2 (NT2) and healthy controls (HC).

	NT1 (*n* = 21) Mean ± SD	NT2 (*n* = 15) Mean ± SD	HC (*n* = 22) Mean ± SD	F_(2,55)_	*p*	NT1 vs NT2 post-hoc	NT1 vs HC post-hoc	NT2 vs HC post-hoc
*Demographic and clinical data*								
Gender (M/F)	9/12	6/9	11/11	0.41 ^a^	*ns*			
Age (years)	36.19 ± 11.94	35.53 ± 12.95	34.95 ± 11.52	0.06 ^b^	*ns*			
BMI	30.41 ± 6.28	25.38 ± 2.86	21.88 ± 3.13	19.48 ^b^	<0.0001	<0.005	<0.0001	*ns*
ESS	15.90 ± 5.31	15.53 ± 5.45	7.05 ± 3.34	23.45 ^b^	<0.0001	*ns*	<0.0001	<0.0001
HLA-DQB1*0602 positivity	19/20	6/12		8.89 ^a^	<0.005			
CSF Hcrt-1	23.31 ± 35.29	334.38 ± 95.19 (*n* = 13/15)		7.47 ^d^	<0.0001			
*Neurophysiological data*								
MSLT-sol	4.41 ± 4.80	6.06 ± 1.59		65 ^c^	<0.005			
SOREMPs	3.71 ± 1.49	2.53 ± 1.06		68 ^c^	<0.005			
*Self-report measure*								
Chronotype *m/i/e*	3/13/5	4/10/1	1/18/3	5.56 ^a^	*ns*			
BDI	16.86 ± 13.13	16.27 ± 11.98	8.14 ± 5.60	4.42 ^b^	<0.05	*ns*	<0.05	*ns*
STAI-S	46.33 ± 14.01	44.20 ± 11.92	38.91 ± 9.55	2.19 ^b^	*ns*			
STAI-T	47.81 ± 14.37	47.47 ± 14.23	40.27 ± 9.63	2.31 ^b^	*ns*			
OCIr	18.67 ± 13.25	15.33 ± 10.22	11.32 ± 8.36	2.48 ^b^	*ns*			
ASRS_In_	17.62 ± 6.69	18.47 ± 9.74	10.64 ± 4.07	7.93 ^b^	<0.001	*ns*	<0.005	<0.005
ASRS_Hy_	14.81 ± 5.51	13.80 ± 5.21	10.77 ± 5.02	3.40 ^b^	<0.05	*ns*	<0.05	*ns*

BMI = body mass index; ESS = Epworth Sleepiness Scale; CSF Hcrt-1 = cerebrospinal fluid hypocretin-1; MSLT-sol = mean sleep latency on MSLT; SOREMPs = sleep-onset REM periods on MSLT; Chronotype: *m* = morning type, *i* = intermediate type, *e* = evening type; BDI = Beck Depression Inventory; STAI = State Trait Anxiety Inventory (S = State, T = Trait); OCIr = reduced obsessive-compulsive inventory; ASRS = adult ADHD self-report scale symptom checklist (In = Inattentive, Hy = Hyperactive).

^*a*^ Chi-square test;

^*b*^ One-way ANOVA;

^*c*^ Mann-Whitney U test;

^*d*^ Independent sample *t*-test.

ANOVA revealed a main group effect for ASRS_In_, with NT1 and NT2 reporting more severe ADHD inattentive symptoms, and NT1 also showing more severe ADHD hyperactive/impulsive and depressive symptoms than controls, without any further between-group difference.

ANOVA revealed a main group effect for ASRS_In_, with NT1 and NT2 reporting more severe ADHD inattentive symptoms, and NT1 also showing more severe ADHD hyperactive/impulsive and depressive symptoms than controls, without any further between-group difference. Classifying ASRS_Scr_ score into four-stratum (0–24 scoring) and applying the cut-off of 14 proposed by Kessler et al. [30], 33.33% and 26.66% of NT1 and NT2 patients displayed scores in the pathological range (i.e. 14–24 interval), while all controls fell into the normal range (i.e. 0–13 interval). Despite a trend towards higher anxiety levels in the clinical groups, differences in state and trait anxiety levels and OCIr score did not reach statistical significance.

### 3.2 Attention network test

**Fig 1:**
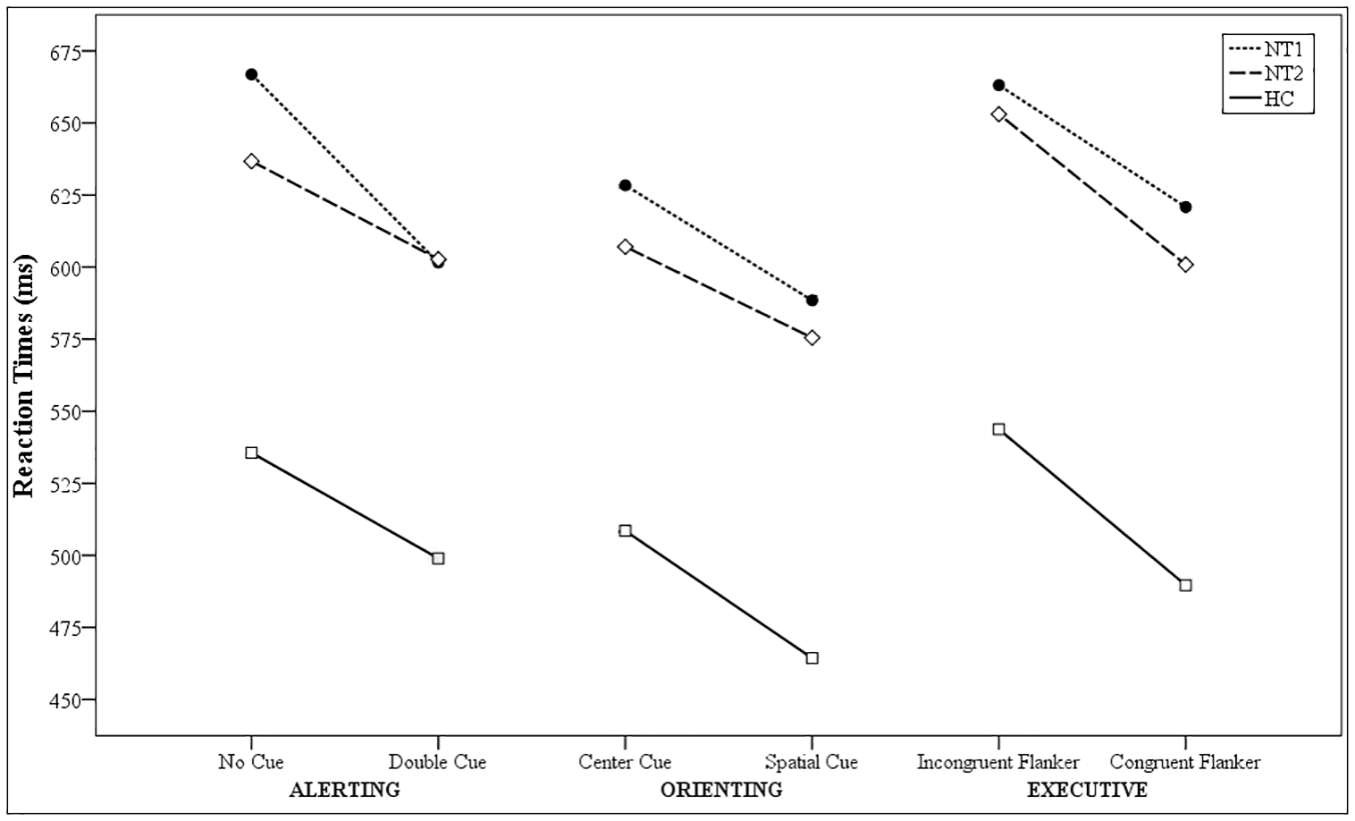
Mean reaction times (ms) for the cue and flanker conditions relevant for each attentional network.

Mean and standard deviation of ANT network absolute and proportional scores (Pro), overall RTs, and accuracy are reported in Table 2, together with significance values at ANOVA, *η*^2^_*p*_, and post-hoc results. NT1 and NT2 displayed slower overall RTs compared to controls, without differing in performance accuracy. NT1 patients showed higher alerting network absolute and proportional scores compared to NT2 patients and controls. Conversely, no differences emerged between patients and controls on orienting and executive networks absolute and proportional scores. Results of the three mixed ANOVA on mean RTs for the cues and flankers relevant for the attentional processes are shown in Fig 1.

**Table 2:** Mean, standard deviation (SD), univariate and post-hoc results of attentional network absolute and proportional score (Pro) of narcolepsy type 1 (NT1), narcolepsy type 2 (NT2) and healthy controls (HC).

	NT1 (*n* = 21) Mean ± SD	NT2 (*n* = 15) Mean ± SD	HC (*n* = 22) Mean ± SD	Univariate Results			NT1 vs NT2	NT1 vs HC	NT2 vs HC
				F_(2,55)_	*p*	*η*^2^_*p*_	Bonferroni Post-Hoc	Bonferroni Post-Hoc	Bonferroni Post-Hoc
Alerting	65.30 ± 35.17	34 ± 27.22	33.44 ± 18.90	8.65	<0.001	.239	<0.005	<0.001	
Alerting (Pro)	0.11 ± 0.06	0.06 ± 0.05	0.07 ± 0.04	5.44	<0.01	.165	<0.05	<0.05	
Orienting	39.92 ± 22.77	31.57 ± 29.21	44.17 ± 29.33	0.97	*ns*				
Orienting(Pro)	0.06 ± 0.03	0.05 ± 0.05	0.09 ± 0.06	2.76	*ns*				
Executive	42.28 ± 30.17	52.19 ± 37.61	54.07 ± 32.01	0.77	*ns*				
Executive(Pro)	0.07 ± 0.05	0.08 ± 0.05	0.11 ± 0.07	2.31	*ns*				
Overall RTs	621.08 ± 111.28	605.09 ± 123.01	501.60 ± 52.26	9.37	<0.0005	.254		<0.001	<0.01
Accuracy(%)	97.56 ± 2.5	96.52 ± 5.03	98.82 ± 1.06	2.68	*ns*				

#### 3.2.1 ANT–alerting effect

The alerting effect was explored with a 3 (group: NT1, NT2, controls) X 2 (cue condition: no-cue, double-cue) mixed ANOVA. A main effect of group (F_2,55_ = 8.960; *p* < 0.005; *η*^2^_*p*_ = 0.246) with both NT1 and NT2 patients responding more slowly than controls, and a main effect of cue (F_1,55_ = 143.346; *p* < 0.0001; *η*^2^_*p*_ = 0.721) with faster RTs in the double-cue than in the no-cue condition were found. The two-way interaction cue condition by group was statistically significant (F_2,55_ = 8.65; *p* < 0.001; *η*^2^_*p*_ = 0.239); this interaction was due to larger differences between RTs in no-cue (666.86 ± 115.93 ms) relative to double-cue (601.57 ± 110.23 ms) condition of NT1 patients compared to NT2 (no-cue 636.73 ± 126.03 ms, double-clue 602.73 ± 122.53 ms) and controls (no-cue 533.77 ± 56.53 ms, double-clue 500.32 ± 51.81 ms).

#### 3.2.2 ANT–orienting effect

The orienting effect was explored with a 3 (group: NT1, NT2, controls) X 2 (cue condition: center-cue, spatial-cue) mixed ANOVA. A main effect of group (F_2,55_ = 9.727; *p* < 0.001; *η*^2^_*p*_ = 0.261) with both clinical groups being slower than controls, and a main effect of cue (F_1,55_ = 114.074; *p* < 0.0001; *η*^2^_*p*_ = 0.675) with faster RTs in the spatial-cue compared to center-cue condition were found. The interaction between factors did not reach statistical significance (F_2,55_ = 0.971; *p* = 0.385).

#### 3.2.3 ANT–executive control

The flanker effect was explored with a 3 (group: NT1, NT2, controls) X 2 (flanker type: congruent, incongruent) mixed ANOVA. A main effect of group (F_2,55_ = 9.232; *p* < 0.001; *η*^2^_*p*_ = 0.251) with both clinical groups being slower than controls, and a main effect of flanker (F_1,55_ = 127.648; *p* < 0.0001; *η*^2^_*p*_ = 0.699) with faster RTs in the congruent-flanker compared to incongruent-flanker were found. The interaction between factors (F_2,55_ = 0.767; *p* = 0.469) did not reach statistical significance.

### 3.3 Correlation analyses

Pearson’s correlation analyses are reported separately for each group in Table 3. In the NT1 group the ESS was directly related to ASRS_In_ and inversely related to alerting network score, while the BDI was positively correlated with ESS, ASRS_In_ and ASRS_Hy_. In the NT2 group, only a positive correlation between ASRS_In_ and ASRS_Hy_ reached statistical significance. No significant correlations were observed in the control group.

**Table 3:** Correlations between attentional network score and self-reported measures of depression, sleepiness, ADHD inattentive and hyperactive symptoms.

	NT1 (*n* = 21)				NT2 (*n* = 15)				HC (*n* = 22)			
	ESS	BDI	ASRS_In_	ASRS_Hy_	ESS	BDI	ASRS_In_	ASRS_Hy_	ESS	BDI	ASRS_In_	ASRS_Hy_
Alerting	-0.56**	-0.32	-0.16	0.12	0.32	-0.41	-0.28	-0.05	0.01	0.06	0.15	0.11
Orienting	0.14	0.06	0.04	-0.24	0.01	-0.10	-0.29	-0.23	0.05	-0.26	-0.39	-0.18
Executive	-0.03	0.40	-0.04	0.33	0.42	-0.15	-0.26	-0.24	0.05	-0.20	-0.10	0.10
ESS	**-**	0.55**	0.47*	0.17	**-**	-0.42	-0.33	-0.09	**-**	-0.01	-0.07	-0.35
BDI		**-**	0.67**	0.52*		**-**	0.30	0.38		**-**	0.39	0.27
ASRS_In_			**-**	0.46*			**-**	0.77**			**-**	0.26
ASRS_Hy_				**-**				**-**				**-**

NT1 = narcolepsy type 1; NT2 = narcolepsy type 2; HC = healthy controls; ESS = Epworth Sleepiness Scale; BDI = Beck Depression Inventory; ASRS = adult ADHD self-report scale symptom checklist (In = Inattentive, Hy = Hyperactive).

* *p* <0.05

** *p* <0.01

## Discussion

Our study is the first to investigate attention networks functioning [16], by means of the ANT, in adult drug-naïve NT1 and NT2 patients, and assessing in parallel self reported ADHD, OCD, anxiety and depression symptoms. We found a specific alteration of the alerting network in NT1, mirrored by a greater alerting effect, while no group differences were observed in the orienting and executive network. In line with previous studies, NT1 and NT2 patients had slower RTs than controls, without differing in performance accuracy [9,14,15]. Differences in the alerting network emerged also in the proportional scores, proving that such impairment is independent from the global slowing [35].

Given that the ANT network scores are computed with a subtractive logic, a greater alerting effect does not necessarily indicate less efficient performances, but may mirror the need to compensate difficulties in performing the task by increasing the effort [37], as suggested by the negative correlation with the ESS.

In this scenario, it is crucial to consider the distinction between tonic and phasic components of alertness process: in trials without warning cue participants rely exclusively on their internal arousal system and RTs reflects tonic alertness. Conversely, when a warning cue precedes the target, participants use this information to speed-up RTs and RTs reflects phasic alertness response (i.e. arousability). Since RTs in trials with double-cues are similar between NT1 and NT2 compared to trials without warning cues, we conclude that in NT1 tonic alertness is markedly impaired while phasic alertness reaction, while performing a short task, is essentially preserved. This attentional profile of impaired tonic alertness and preserved arousability is consistent with previous studies on attentional functioning in NT1: in an extensive investigation, Rieger et al. reported specific deficits in divided and flexible attention, with preserved phasic alertness and focused attention [14]. Similarly, Naumann et al. reported preserved phasic alertness reaction in NT1, while RTs slowing emerged in tasks assessing divided attention and working memory [9]. In contrast with previous reports, no deficit in the executive control of attention was observed [13,15,38]. Noteworthy, a similar attentional profile with greater alerting effect and preserved orienting and executive networks has been highlighted in children with ADHD inattentive subtype [39].

The ANT proved reliable in different clinical settings, by linking specific attentional dysfunctions with well-grounded anatomical and biochemical deficits [40,41]. Neuroimaging studies showed that the alerting network depends on the activation of the frontal and parietal area and its functions are modulated by the noradrenergic system arising from the locus coeruleus [16,42]. The hypocretinergic system has strong projections to the locus coeruleus noradrenergic system and a reduced stimulation may account for the abnormal alerting network functioning highlighted in NT1 [5]. A recent positron emission tomography study in NT1 is in line with our findings showing significantly reduced metabolism in the frontal regions [43].

Regarding self-reported measures, narcolepsy patients share more severe ADHD inattentive symptoms, while NT1 patients also had more severe hyperactive-impulsive and depressive symptoms than controls.

We only found a non-significant trend towards higher anxiety levels and OCD symptoms in NT1 and NT2 compared to controls; previous studies reported conflicting results with a higher [20,21] and comparable [44] anxiety level in NT1 compared to healthy controls, while OCD domain has never been investigated.

The more severe ADHD inattentive symptoms in NT1 and NT2 patients might be influenced by or in overlap with sleepiness, as partly suggested by correlation analyses. Conversely, NT1 and NT2 patients did not differ in the level of subjective sleepiness and only NT1 patients showed more severe ADHD hyperactive-impulsive symptoms pointing to a direct role of hypocretin in modulating impulsivity. The proportion of patients showing clinically significant adult ADHD symptoms is 33.33% for the NT1 group and 26.66% for the NT2 group compared with 0% of healthy controls.

Our results are in line with previous investigations that reported an association between ADHD symptomatology and narcolepsy in NT1 children [22,23], and highlight, for the first time, that an overrepresentation of clinically-significant ADHD symptoms may persist in adult NT1 cases.

Nevertheless, caution should be used in interpreting these data since they come from questionnaires and not from a clinical diagnosis of ADHD.

Our study also has limitations. First, the ANT considers as a proxy of executive functions the ability to resolve conflicts in the processing of competing stimuli: although the latter is a key aspect of executive functions, this multifaceted cognitive domain encompasses a wide set of higher-order cognitive processes including inhibition, set-shifting, multitasking, planning and working memory. It is therefore possible that NT1 patients present with more difficulty in executive functions than those assessed by the ANT.

Second, the sample size is relatively small and further studies are required to confirm our results. Further studies are required to investigate the activation of attentional networks in NT1 by fMRI while performing the ANT and to investigate whether the ANT could be a reliable tool to assess the effects of pharmacological treatments on attentional profile in NT1.

In conclusion, our study showed that the attentional profile in narcolepsy is objectively characterized by an overall slowing and by a further selective deficit in the alerting network in hypocretin deficient patients (NT1). The relevance of this profile is mirrored by the clinically significant association with the inattentive and hyperactive ADHD symptoms. As a practical consequence, physicians should actively address not only sleep-related symptoms, but also attentional impairment and psychopathological symptoms to better understand disease burden.

## Supporting information

S1 Dataset Demographic, clinical, questionnaire and ANT data of participants. (XLSX)

## Abbreviations

ADHD: Attention-deficit/hyperactivity disorder
ANT: Attention network test
ASRS_Hy_: Adult ADHD Self-Report Scale Symptom Checklist, hyperactive score
ASRS_In_: Adult ADHD Self-Report Scale Symptom Checklist, inattentive score
BDI: Beck Depression Inventory
BMI: Body mass index
CFS: Cerebrospinal fluid
ESS: Epworth sleepiness scale
Hcrt-1: Hypocretin-1
HLA: Human leukocyte antigen
ICSD-3: International classification of sleep disorders 3^rd^ edition
MSLT: Multiple sleep latency test
NT1: Narcolepsy type 1
NT2: Narcolepsy type 2
OCD: Obsessive-compulsive disorder
OCIr: Obsessive Compulsive Inventory-Revised
Pro: Attention network proportional score
REM: Rapid eye movement
rMEQ: Reduced morningness-eveningness questionnaire
RTs: Reaction times
SOREMPs: MSLT sleep-onset REM periods
STAI: State-Trait Anxiety Inventory
*η*^2^_*p*_: Partial eta squared
